# Performance Evaluation and Implications of Large Language Models in Radiology Board Exams: Prospective Comparative Analysis

**DOI:** 10.2196/64284

**Published:** 2025-01-16

**Authors:** Boxiong Wei

**Affiliations:** 1 Department of Ultrasound, Peking University First Hospital, 8 Xishiku Rd, Xicheng District, Beijing, 100034, China, 86 13132150190, 86 314521

**Keywords:** large language models, LLM, artificial intelligence, AI, GPT-4, radiology exams, medical education, diagnostics, medical training, radiology, ultrasound

## Abstract

**Background:**

Artificial intelligence advancements have enabled large language models to significantly impact radiology education and diagnostic accuracy.

**Objective:**

This study evaluates the performance of mainstream large language models, including GPT-4, Claude, Bard, Tongyi Qianwen, and Gemini Pro, in radiology board exams.

**Methods:**

A comparative analysis of 150 multiple-choice questions from radiology board exams without images was conducted. Models were assessed on their accuracy for text-based questions and were categorized by cognitive levels and medical specialties using *χ*^2^ tests and ANOVA.

**Results:**

GPT-4 achieved the highest accuracy (83.3%, 125/150), significantly outperforming all other models. Specifically, Claude achieved an accuracy of 62% (93/150; *P*<.001), Bard 54.7% (82/150; *P*<.001), Tongyi Qianwen 70.7% (106/150; *P*=.009), and Gemini Pro 55.3% (83/150; *P*<.001). The odds ratios compared to GPT-4 were 0.33 (95% CI 0.18‐0.60) for Claude, 0.24 (95% CI 0.13‐0.44) for Bard, and 0.25 (95% CI 0.14‐0.45) for Gemini Pro. Tongyi Qianwen performed relatively well with an accuracy of 70.7% (106/150; *P*=0.02) and had an odds ratio of 0.48 (95% CI 0.27‐0.87) compared to GPT-4. Performance varied across question types and specialties, with GPT-4 excelling in both lower-order and higher-order questions, while Claude and Bard struggled with complex diagnostic questions.

**Conclusions:**

GPT-4 and Tongyi Qianwen show promise in medical education and training. The study emphasizes the need for domain-specific training datasets to enhance large language models’ effectiveness in specialized fields like radiology.

## Introduction

Artificial intelligence (AI) in radiology has significantly improved diagnostic accuracy and educational methods for radiologists. By using advanced machine learning and deep learning techniques, AI applications have evolved from enhancing image interpretation to supporting complex diagnostic decisions [1]. These advancements not only increase the efficiency of diagnostic processes but also provide radiologists with interactive training simulations, crucial for their professional growth and certification readiness [2-9].

Recent advancements have also emerged with the development of large language models (LLMs) like GPT-4, Claude, Bard, Tongyi Qianwen and Gemini Pro. These models have added a new aspect to medical education by producing medically accurate content and supporting advanced diagnostic reasoning exercises [10,11]. These features are crucial for establishing safe learning spaces where future radiologists can practice detailed diagnostic reasoning and decision-making without real-world clinical risks [12,13]. Moreover, these LLMs are crucial in developing and clarifying complex medical scenarios and test questions, improving the educational experience and boosting the diagnostic abilities of students [14-16].

Despite these advancements, recent research has pinpointed limitations in the use of LLMs in medical exams, particularly in specialties like radiology that demand extensive clinical insight. Studies have shown that while LLMs such as GPT-4 can manage simple diagnostic questions effectively, they encounter difficulties with more complex cases that require a deeper clinical understanding and the integration of diverse medical information [17,18]. These findings highlight a significant gap in the existing literature; there is a lack of comprehensive comparative studies that evaluate the performance of various LLMs across different diagnostic scenarios in radiology [19].

This study addresses this gap by comparing several mainstream LLMs in text-based radiology board exams, without imaging components, evaluating their overall performance. While a secondary objective is to analyze performance by question type and topic. This study hypothesizes that GPT-4 will outperform other models, particularly in handling complex diagnostic questions.

## Methods

### Study Design

This research was structured as a prospective, comparative analysis that aimed to test the effectiveness of various notable LLMs within a controlled environment resembling radiology board examinations without images. The radiology exams comprehensively evaluated a candidate’s radiology knowledge, reasoning, and clinical skills. China does not currently have a unified national licensing exam specifically for radiologists. Given that the Canadian Royal College and American Board of Radiology exams are viewed as authoritative and widely recognized, test questions were selected according to the standards of these two exams for model testing [20]. Both of the exams assess candidates on a broad spectrum of radiology topics using multiple-choice questions.

### Ethical Considerations

Despite the reliance on nonpersonal, pre-existing data and the lack of direct involvement of human or animal subjects, ethical approval and the need for informed consent were waived by the Institutional Review Board of Peking University First Hospital, Beijing, China. The radiologists who participated in question validation and categorization were compensated at a rate of 300 Chinese Yuan (US $40.91) per hour for their professional expertise. All data used in the study were anonymized exam questions, with no personal identifiable information involved. The research strictly adhered to ethical standards, with data integrity meticulously upheld throughout the study.

### Models Selection

The models chosen for this investigation included GPT-4 (OpenAI), Claude 2.1 (Anthropic), Bard (Google, PaLM 2), Tongyi Qianwen (Alibaba, Qwen-72B), and Gemini Pro 1.0 (Google). All models were tested from late November to early December 2023. These models represent significant advancements in AI, particularly in natural language processing. They were selected based on their demonstrated success in academic and professional settings, indicating their potential effectiveness in educational applications.

### Dataset Composition

The dataset for this study consisted of 150 multiple-choice questions drawn from historical radiology board exams similar to those given by the Canadian Royal College and the American Board of Radiology. These questions were sourced from the websites of Board Vitals [21] and CanadaQBank [22], which are widely recognized for providing questions that closely reflect the content and format of North American radiology board exams. Each question was individually reviewed and validated by two academic radiologists—one specializing in ultrasound with 20 years of experience and the other in abdominal radiology with 4 years of experience. Questions were only included if both reviewers concurred on their relevance and appropriateness for this study. Questions that involved images were excluded.

### Question Categorization

All questions were classified according to their primary assessment objectives using Bloom’s Taxonomy, including two main categories: lower-order thinking (remembering and understanding) and higher-order thinking (applying, analyzing, and evaluating) [23]. Higher-order thinking questions were further divided into specific groups such as description and analysis of image findings, application of concepts, clinical management, and calculation and classification. Additionally, questions were also classified based on the specific area of disease focus, including digestive, genitourinary, musculoskeletal, respiratory, cardiovascular (including angiography and intervention), nervous, breast and thyroid, pediatrics, and imaging basics and physics. Each question was reviewed and categorized independently by the two board-certified radiologists mentioned above. Any disagreements were then discussed collectively to arrive at a consensus.

### Scoring Criteria

The Canadian Royal College examination uses a pass-fail system based on achieving at least 70% on all written components of the examination. The American Board of Radiology uses a criterion-referenced scoring system. This means that candidates are evaluated against a predefined standard, not in comparison to other test-takers. The passing standard is typically set by a group of experts, including residency program directors and experienced clinicians, who determine the difficulty level of each question to ensure it aligns with the required competency for independent practice. To pass, candidates must meet or exceed the passing standard for all categories scored together. For both exams, the questions undergo psychometric validation, and questions that are not effective in discriminating between candidates or are found too difficult may be removed. The threshold for passing in this study was set at 70% to align with the standards of the Royal College examinations in Canada. This study did not use the criterion-referenced scoring system used by the American Board of Radiology because its standards were difficult to ascertain. Each multiple-choice question was inputted into different LLMs, and the first response from each model was recorded as the subject of analysis.

### Statistical Analysis

To evaluate the association between model type and accuracy for categorical variables, *χ*^2^ tests were used. For categories with small sample sizes, the Fisher exact test was used to ensure the validity of the statistical results. Odds ratios and their corresponding 95% CIs were calculated using GPT-4 as the benchmark. ANOVA was used to compare the mean accuracy rates across different models. Following the results from the ANOVA, Tukey’s honestly significant difference test was applied to identify specific pairs of models that demonstrated significant differences in performance. Cohen *d* was calculated to quantify the magnitude of differences between the models, providing a clearer understanding of the practical significance of the findings. Split-half reliability testing was used to assess the consistency of each model’s performance across different subsets of data, ensuring the reliability of the models over varied test conditions. Statistical significance was set at an α level of .05.

## Results

### Overall Model Performance

GPT-4 emerged as the leading model with an accuracy rate of 83.3% (125/150), significantly outperforming its peers. Tongyi Qianwen also displayed strong performance, recording a 70.7% (106/150) accuracy. Moderate effectiveness was observed in models like Claude and Gemini Pro, with accuracy rates of 62.0% (93/150) and 55.3% (83/150), respectively. Bard trailed with a 54.7% (82/150) accuracy rate, highlighting its challenges in handling complex medical data under exam conditions (Table 1).

**Table 1.** Performance of different large language models on radiology board–styled multiple-choice questions without images.

Parameter	Test score, n (%)				
	GPT4	Claude	Bard	Tongyi Qianwen	Gemini Pro
All questions (n=150)	125 (83.3)	93 (62.0)	82 (54.7)	106 (70.7)	83 (55.3)
Question type					
Lower order thinking (n=46)	38 (82.6)	34 (73.9)	27 (58.7)	34 (73.9)	29 (63)
Higher order thinking (n=104)	87 (83.7)	59 (56.7)	55 (52.9)	72 (69.2)	54 (51.9)
Higher order thinking question categories					
Description and analyze of image findings (n=35)	30 (85.7)	23 (65.7)	20 (57.1)	28 (80)	21 (60)
Application of concepts (n=38)	34 (89.5)	19 (50)	17 (44.7)	26 (68.4)	17 (44.7)
Clinical management (n=19)	14 (73.7)	12 (63.2)	12 (63.2)	13 (68.4)	11 (57.9)
Calculation and classification (n=12)	9 (75)	5 (41.7)	6 (50)	5 (41.7)	5 (41.7)
Question topic					
Digestive (n=15)	10 (66.7)	7 (46.7)	5 (33.3)	10 (66.7)	9 (60)
Genitourinary (n=21)	19 (90.5)	15 (71.4)	14 (66.7)	15 (71.4)	11 (52.4)
Musculoskeletal (n=11)	8 (72.7)	6 (54.5)	7 (63.6)	9 (81.8)	7 (63.6)
Respiratory (n=15)	12 (80)	9 (60)	8 (53.3)	8 (53.3)	8 (53.3)
Cardiovascular (n=22)	19 (86.4)	14 (63.6)	8 (36.4)	18 (81.8)	11 (50)
Nervous (n=11)	11 (100)	9 (81.8)	7 (63.6)	8 (72.7)	9 (81.8)
Breast and thyroid (n=14)	11 (78.6)	9 (64.3)	9 (64.3)	9 (64.3)	7 (50)
Pediatrics (n=19)	15 (78.9)	11 (57.9)	11 (57.9)	13 (68.4)	9 (47.4)
Imaging Basics and physics (n=22)	19 (86.4)	11 (50)	12 (54.5)	15 (68.2)	12 (54.5)

### Detailed Performance Analysis by Question Type

**Figure 1.**
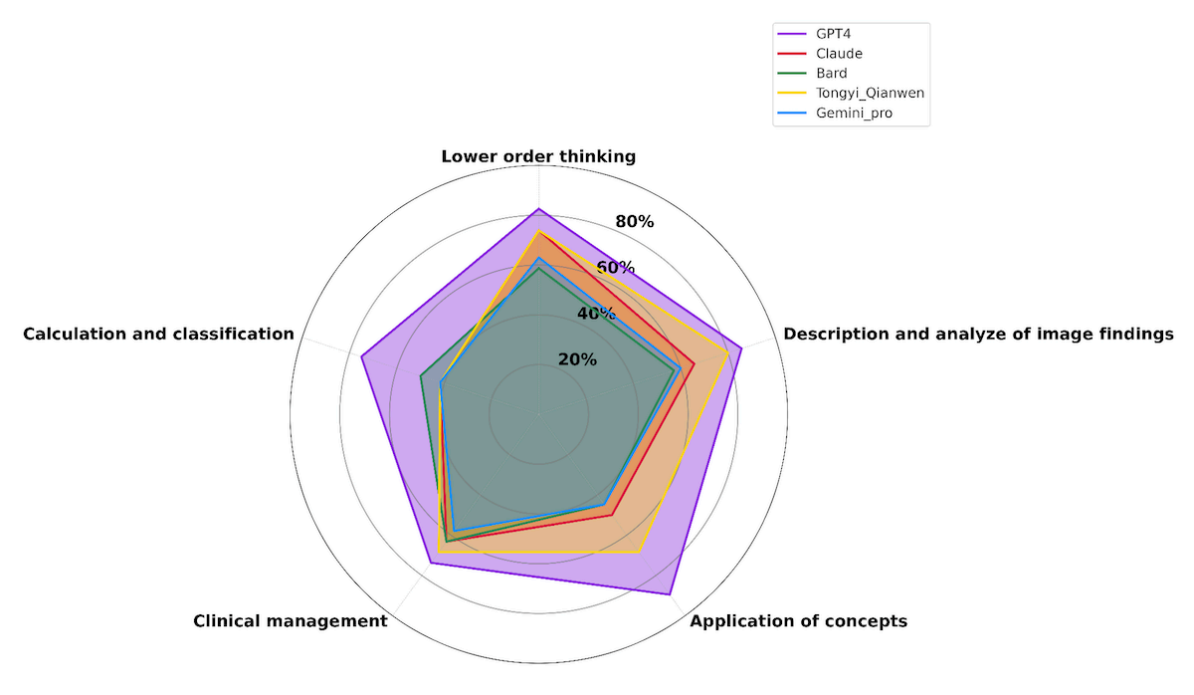
Model accuracy by question type, illustrating the differentiation in model performance between lower-order and higher-order thinking questions.

The breakdown by question type revealed that GPT-4 consistently excelled in both lower-order and higher-order thinking questions, scoring 82.6% (38/46) and 83.7% (87/104), respectively. This indicated GPT-4’s capability to manage both basic recall and more complex analytical tasks effectively. In contrast, models such as Claude and Bard demonstrated a drop in performance with higher-order thinking questions, achieving only 56.7% (59/104) and 52.9% (55/104) accuracy in this category, respectively. This gradient in performance highlighted the difficulties faced by current LLMs in simulating the complex cognitive processes involved in clinical reasoning (Figure 1).

### Performance Across Medical Specialties

Performance analysis segmented by medical specialty showed marked variances. GPT-4 demonstrated exceptional proficiency in neurology with a perfect score of 100% (11/11), and also performed well in genitourinary and cardiovascular categories, with accuracies of 90.5% (19/21) and 86.4% (19/22), respectively. However, challenges were apparent in areas like musculoskeletal and digestive categories, where high-performing models like GPT-4 experienced reduced accuracy rates of 72.7% (8/11) and 66.7% (10/15), respectively. These results indicated that some specialties may need more tailored domain-specific training for models to enhance their effectiveness (Table 1).

Detailed odds ratios and CIs for each model are presented in Multimedia Appendix 1. The odds ratio results show that GPT-4 had the highest performance. All the other models had significantly lower odds ratios compared to GPT-4. Tongyi Qianwen had the highest odds ratio among the other models. As shown in Multimedia Appendix 2, the pairwise comparisons showed that GPT-4 significantly outperformed all other models, with statistically significant differences observed in its comparison with Claude (*P*<.001), Bard (*P*<.001), Tongyi Qianwen (*P*=.009), and Gemini Pro (*P*<.001). Additionally, Tongyi Qianwen exhibited a significantly higher accuracy compared to Bard (*P*=.004) and Gemini Pro (*P*=.006). In contrast, no statistically significant differences were found between Claude and Bard (*P*=.20), Claude and Gemini Pro (*P*=.24), or Bard and Gemini Pro (*P*=.90). These results suggest that the performance of these models was relatively similar in this dataset.

## Discussion

### Principal Findings

The exceptional performance of GPT-4 in this study aligns with recent findings that highlight its advanced reasoning capabilities and improvements over previous versions, such as GPT-3.5, in various professional contexts, including various kinds of medical exams [24]. GPT-4’s extensive training on diverse datasets and its refined architecture enable it to adeptly handle complex questions, which are typical in the specialized language and scenario-based queries found in medical board examinations [25]. Nevertheless, the performance differences observed among models like Bard and Claude can be attributed to the nature of their training and inherent limitations in processing complex cognitive tasks, which are crucial in radiology examinations. This is largely due to the absence of specialized medical training data during their development phases. These findings are in line with the research, which indicated that while GPT-4’s textual reasoning is strong, its integration and analysis of image-based information remains inadequate [26].

Models such as GPT-4 and Tongyi Qianwen, which displayed superior performance, likely benefited from training datasets that included medical scenarios. The significance of domain-specific training is well-documented, emphasizing that for LLMs to excel in specialized fields like radiology, they require training with pertinent medical data. Both GPT-4 and Tongyi Qianwen exceeded the 70% passing threshold for the simulated radiology board exams. This marks a significant achievement and shows the potential of these models in academic and professional environments. The threshold mirrors real medical licensing exam criteria, offering a realistic measure of AI’s potential performance in actual educational assessments. The robust performance of Tongyi Qianwen, particularly in an English-based setup, is notable. Despite generally not being ranked as highly as Western models in AI benchmarks, its performance indicates significant progress in China’s AI development [27]. This supports calls for more inclusive and diverse training datasets to reduce biases and improve the global applicability of AI technologies.

GPT-4 has demonstrated the capability to pass simulated UK Radiology Fellowship Examinations, especially in sections focused on physics and single best answers [28]. However, challenges remain when these models are tested with image-based questions, highlighting a persisting gap between current AI capabilities and the complex demands of radiological diagnostics [26]. While integrating LLMs into medical education and assessments promises transformative changes in how content is delivered and evaluated, there is a risk of excessive reliance on AI. This overdependence could potentially undermine the development of critical thinking and diagnostic skills vital for medical practice [25].

### Limitations

This study’s limitations include its sole focus on text-based questions and the exclusion of visual components, which are integral to radiology. Future research should incorporate multimodal assessments and also aim to integrate image recognition capabilities with textual analysis to improve the applicability of LLMs in radiology. These models will need to be fine-tuned with domain-specific datasets to enhance their practical utility in medical education and clinical diagnostics. Another notable limitation is the delay between the submission and publication of peer-reviewed articles, which can result in outdated assessments of rapidly evolving LLMs. The models evaluated in this paper were based on their versions from late November to early December 2023, and significant advancements have occurred since then, particularly with models like Claude, which has been regularly updated, with multiple new versions released by Anthropic. In future work, we intend to continue discussing the accuracy comparisons among new models as they are released. Additionally, if sufficient technical resources are available, we aim to create a platform to maintain an up-to-date database of LLM performance on this benchmark.

### Conclusion

This article underscores the evolving capabilities and limitations of LLMs in medical education. While models like GPT-4 show promise, the path to their effective integration in clinical practice requires ongoing refinement and a deeper understanding of their operational dynamics in complex medical settings.

## Supplementary material

10.2196/64284 Multimedia Appendix 1 The odds ratios and CIs of each model using GPT-4 as the benchmark.

10.2196/64284 Multimedia Appendix 2 Hypothetical pairwise comparison table.

## Abbreviations

AI: artificial intelligence
LLM: large language model
