# Supplementary material for: Performance Evaluation and Implications of Large Language Models in Radiology Board Exams: Prospective Comparative Analysis
Source: JMIR Med Educ. 2025 Jan 16;11:e64284. doi: 10.2196/64284 (PMC11756834; doi:10.2196/64284)
Supplement: Multimedia Appendix 1 [file mededu-v11-e64284-s001.docx]

Table S1 The Odds Ratios and Confidence Intervals of each model using GPT-4 as the Benchmark.

| Model | Correct Answers | Incorrect Answers | Odds | Odds Ratio (vs GPT-4) | 95% Confidence Interval |
| --- | --- | --- | --- | --- | --- |
| GPT-4 | 125 | 25 | 5 | 1 | - |
| Claude | 93 | 57 | 1.63 | 0.33 | (0.18 - 0.60) |
| Bard | 82 | 68 | 1.21 | 0.24 | (0.13 - 0.44) |
| Tongyi Qianwen | 106 | 44 | 2.41 | 0.48 | (0.27 - 0.87) |
| Gemini Pro | 83 | 67 | 1.24 | 0.25 | (0.14 - 0.45) |
