# Supplementary material for: Performance Evaluation and Implications of Large Language Models in Radiology Board Exams: Prospective Comparative Analysis
Source: JMIR Med Educ. 2025 Jan 16;11:e64284. doi: 10.2196/64284 (PMC11756834; doi:10.2196/64284)
Supplement: Multimedia Appendix 2 [file mededu-v11-e64284-s002.docx]

Table S2 Hypothetical Pairwise Comparison Table

| Comparison | p-value |
| --- | --- |
| GPT-4 vs. Claude | <0.001 |
| GPT-4 vs. Bard | <0.001 |
| GPT-4 vs. Tongyi Qianwen | 0.009 |
| GPT-4 vs. Gemini Pro | <0.001 |
| Claude vs. Bard | 0.198 |
| Claude vs. Tongyi Qianwen | 0.112 |
| Claude vs. Gemini Pro | 0.241 |
| Bard vs. Tongyi Qianwen | 0.004 |
| Bard vs. Gemini Pro | 0.905 |
| Tongyi Qianwen vs. Gemini Pro | 0.006 |
